# Sex-linked markers in the North American green frog (*Rana clamitans*) developed using DArTseq provide early insight into sex chromosome evolution

**DOI:** 10.1186/s12864-016-3209-x

**Published:** 2016-10-28

**Authors:** Max R. Lambert, David K. Skelly, Tariq Ezaz

**Affiliations:** 1 School of Forestry and Environmental Studies, Yale University, Greeley Memorial Lab, 370 Prospect St, New Haven, CT 06511 USA; 2 Institute for Applied Ecology, University of Canberra, Canberra, ACT Australia

**Keywords:** Complexity reduction, GSD, Illumina, RADseq, Sex chromosomes, Sex determination, Sex reversal

## Abstract

**Background:**

The extent to which sex reversal is associated with transitions in sex determining systems (XX-XY, ZZ-ZW, etc.) or abnormal sexual differentiation is predominantly unexplored in amphibians. This is in large part because most amphibian taxa have homomorphic sex chromosomes, which has traditionally made it challenging to identify discordance between phenotypic and genetic sex in amphibians, despite all amphibians having a genetic component to sex determination. Recent advances in molecular techniques such as genome complexity reduction and high throughput sequencing present a valuable avenue for furthering our understanding of sex determination in amphibians and other taxa with homomorphic sex chromosomes like many fish and reptiles.

**Results:**

We use DArTseq as a novel approach to identify sex-linked markers in the North American green frog (*Rana clamitans melanota*) using lab-reared tadpoles as well as wild-caught adults from seven ponds either in undeveloped, forested habitats or suburban ponds known to be subject to contamination by anthropogenic chemicals. The DArTseq methodology identified 13 sex-linked SNP loci and eight presence-absence loci associated with males, indicating an XX-XY system. Both alleles from a single locus show partial high sequence homology to Dmrt1, a gene linked to sex determination and differentiation throughout Metazoa. Two other loci have sequence similarities to regions of the chimpanzee and human X-chromosome as well as the chicken Z-chromosome. Several loci also show geographic variation in sex-linkage, possibly indicating sex chromosome recombination. While all loci are statistically sex-linked, they show varying degrees of female heterozygosity and male homozygosity, providing further evidence that some markers are on regions of the sex chromosomes undergoing higher rates of recombination and therefore further apart from the putative sex determining locus.

**Conclusion:**

The ease of the DArTseq platform provides a useful avenue for future research on sex reversal and sex chromosome evolution in vertebrates, particularly for non-model species with homomorphic or cryptic or nascent sex chromosomes.

**Electronic supplementary material:**

The online version of this article (doi:10.1186/s12864-016-3209-x) contains supplementary material, which is available to authorized users.

## Background

Sex determination and environmental influences on sexual differentiation in amphibians are long-standing interests of biologists for over a century [1–3]. To date, all amphibians are known to have genotypic sex determination (GSD), although the mode of sex determination (i.e., XX-XY or ZZ-ZW) has repeatedly switched throughout amphibian evolutionary history [4, 5]. Some amphibian species even show substantial geographic variation in sex determining modes [2, 6–9]. Despite GSD, numerous laboratory experiments show that amphibian sexual differentiation is sensitive to environmental factors such as temperature [10, 11] or chemical exposure [12, 13]. Taxa with GSD and which are subject to environmental effects on sexual differentiation can exhibit sex reversal, whereby the genotypic sex does not match the phenotypic sex [14]. Often, sex reversal is inferred in laboratory experiments by sex ratio biases [12, 15, 16]. Recently, some studies been able to provide evidence of discordance between sexual genotype and phenotype in response to environmental factors like the pesticide atrazine [13] or the synthetic estrogen 17α-ethynylestradiol (EE2) [3].

While laboratory evidence of sex reversal is sparse, there is a deeper dearth of evidence demonstrating sex reversal in wild populations. To our knowledge, only one study has shown sex reversal in a wild amphibian population. An analysis of three sex-linked microsatellites in the European common frog (*Rana temporaria*) showed that ~10 % of genotypic females had a male phenotype in a single area [14]. However, the environmental context, natural or anthropogenically-impacted, was not discussed. Recent evidence shows that green frog (*Rana* = *Lithobates clamitans melanota*) sex ratios are male-biased at metamorphosis in undeveloped, forested ponds and become more feminized as ponds become increasingly surrounded by residential suburban land use [16]. Green frog populations in suburban ponds also exhibit high frequencies of feminized testes, a condition that is absent in forested ponds [17, 18]. Feminized testes are also common in *R. clamitans* from agricultural areas [19]. Whether sex ratio variation and feminized testes are due to sex reversal in this species is currently unknown and requires sex-linked molecular markers for understanding underlying molecular mechanisms as well as mechanisms of sex reversal. However, ascertaining the genetic sex of most amphibian taxa is challenging at the chromosomal level as most taxa have homomorphic sex chromosomes [20].

Restriction site-associated DNA sequencing (RADseq) has been proposed as a method for developing sex-linked markers in taxa with homomorphic sex chromosomes [21] and has successfully been used to develop sex-linked markers and infer the sex determining mode for several squamate and fish species [21–24]. Recently RADseq has been used to study sex determination in two European anuran species, including an individual family for the European tree frog (*Hyla arborea*) [25] as well as *R. temporaria* [26]. While a linkage map using RADseq produced single nucleotide polymorphisms (SNPs) elucidating the sex chromosomes in *H. arborea* [25], no sex-linked SNPs were identified in the *R. temporaria* from Switzerland [26]. One reason for not discovering any sex-linked markers might have been due to the limited genetic diversity used in the analysis [26].

Here, we use DArTseq™ (Diversity Arrays Technology) which employs a combination of genome complexity reduction and next generation sequencing [27] similar to RADseq [28] or genotyping-by-sequencing (GBS) methods [29]. Our primary goal was to develop a series of markers that are sex-linked in *R. clamitans*, a species putatively experiencing sex reversal in the wild. Rather than constructing linkage maps from a single mating, we performed DArTseq on lab-reared tadpoles from several clutches as well as a diversity of wild-caught adults from multiple populations and across a land use gradient.

## Methods

### Specimen selection

**Fig. 1:**
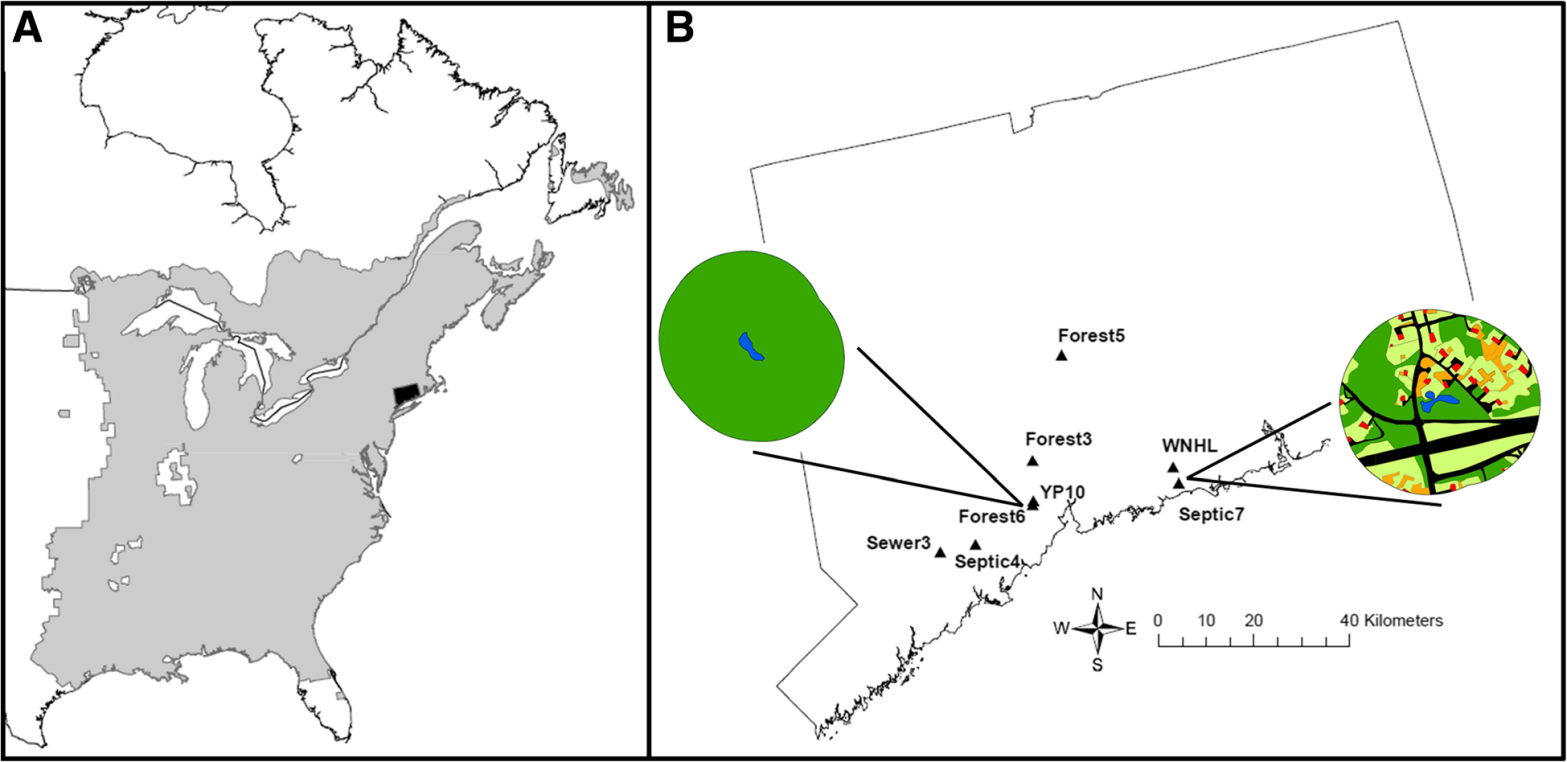
The range (**a**) of *R. clamitans* in North America with the state of Connecticut in *black*. A map (**b**) of the nine source ponds in Connecticut used in this study. Enlarged are two ponds, one forested (Forest6) and one suburban (Septic7), and their land cover within a 200 m buffer of the pond perimeter. *Dark green* = Forest, *Black* = Paved Surface, *Light Green* = Lawn, *Red* = Buildings, *Orange* = Miscellaneous Yard Features. Range data for *R. clamitans* provided by IUCN

**Fig. 2:**
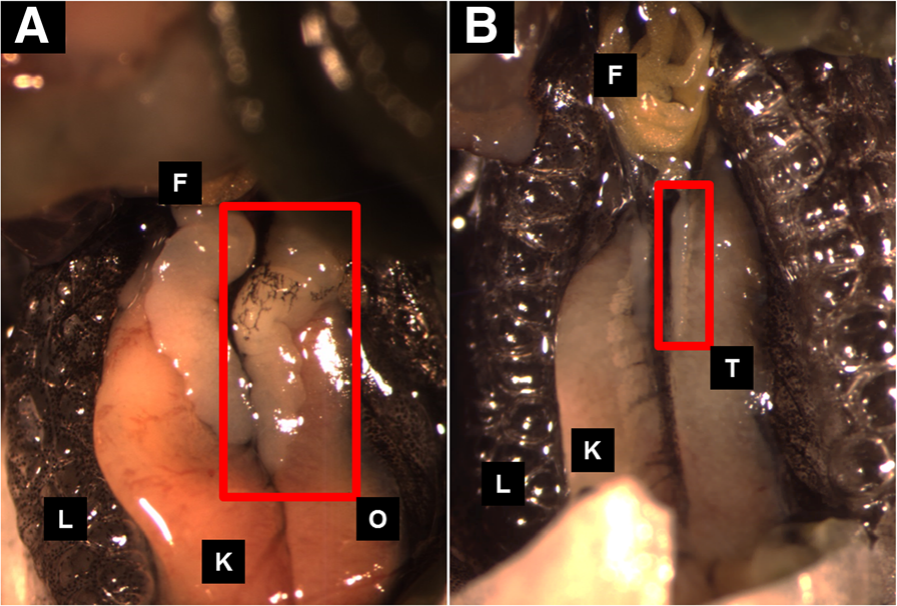
Gonads of 117 day old female (**a**) and male (**b**) from the same clutch collected from pond Forest5. The left ovary and testis are in *red boxes* in each panel. The clutch was collected 06 July, 2015; tadpoles were reared in outdoor mesocosms, and were sampled on 31 October, 2015. *O* = ovary, *T* = testis, *F* = fat body, *K* = kidney, and *L* = lung

The range of *R. clamitans* occupies much of eastern North America, extending from north Florida into southeastern Canada (Fig. 1a); our sampling was located in Connecticut, a state in the northeastern United States (Fig. 1a, b). We used two developmental stages: tadpoles reared to sexual differentiation in controlled-environment mesocosms and wild-caught adults. We predict that tadpoles reared in carefully constructed mesocosms should experience minimal sex reversal, maximizing the probability of genotype-phenotype concordance [30]. We collected portions of freshly-laid clutches from three ponds (1 from Septic7, 3 from Forest5, and 2 from Forest6; Fig. 1b), hatched the embryos in the lab at 19 °C on a 12L: 12D cycle, and reared tadpoles in outdoor mesocosms. We did not use any leaf litter to our mesocosms as leaf leachate has been shown to influence sex steroid pathways [31]. Tadpoles of this species can often be sexed within a few months of hatching despite taking over a year to metamorphose [32]. At ~4 months old, we euthanized tadpoles, sexed them under a dissecting microscope, and collected tail muscle tissue in 90 % ethanol for genetic analysis. Not all tadpoles were fully sexually differentiated at this point and so we focused only on tadpoles with clearly differentiated testes and ovaries (Fig. 2). While not random, we selected tadpoles that had clearly sexually differentiated under controlled conditions. We used 1–3 tadpoles of each phenotypic sex from each clutch for analyses. For adults, we capitalized on Yale Peabody Museum tissues (Additional file 1: Table S1) preserved from wild-caught adults in south-central Connecticut in 2014 as well as an additional five adults from Sewer3 pond collected in 2015. These individuals made up the majority of our specimens and represented variation in local geography and human land use (Fig. 1b). While wild-caught adults may have experienced sex reversal, our goal was to test whether DArTseq could identify sex-linked markers despite the presence of possible sex-reversed individuals and whether we could identify sex-reversed individuals as outliers in our data. We used 1–5 females and 3–11 males from each pond. We used 17 samples in duplicate for sequencing quality control. The source ponds for all specimens ranged from less than 1 km to over 52 km apart (Table 1).

**Table 1:** Pairwise geographic distances (meters) between source ponds

	Septic7	Forest5	Septic4	Forest3	Sewer3	WNHL	YP10	Forest6
Septic7	0	36,081	44,753	31,080	52,290	3519	30,836	31,184
Forest5	36,081	0	43,348	22,776	48,315	32,851	30,782	31,650
Septic4	44,753	43,348	0	21,181	7555	44,647	15,273	14,592
Forest3	31,080	22,776	21,181	0	27,224	29,533	8266	9102
Sewer3	52,290	48,315	7555	27,224	0	52,126	22,381	21,779
WNHL	3519	32,851	44,647	29,533	52,126	0	30,197	30,633
YP10	30,836	30,782	15,273	8266	22,381	30,197	0	871
Forest6	31,184	31,650	14,592	9102	21,779	30,633	871	0

### Genotyping-by-sequencing (GBS)

DNA extractions and sequencing were performed using DArTseq™ (Diversity Arrays Technology Pty Ltd). DArTseq™ represents a combination of a DArT complexity reduction methods and next generation sequencing platforms [27]. Therefore, DArTseq™ represents a new implementation of sequencing of complexity reduced representations [33] and more recent applications of this concept on the next generation sequencing platforms [28, 29], conceptually similar to double digest RADseq. A detailed description of the DArTseq™ methodology can be found in Kilian et al. [27]. While RADseq typically yields markers 85 bp or longer (e.g., [21, 23, 24]), DArTseq™’s criteria (see below) often produce sequences 69 bp or shorter in length. Compared to other similar approaches, DArTseq™ yields a lower density of markers (10’s of thousands and up to 35,000 loci versus >800,000 loci with a GBS approach) but has substantially higher coverage and results in less missing data [34]. In addition to these advantages of DArTseq™, we chose this platform because it can directly score samples as heterozygous/homozygous at each locus with the lower density approach [34] and due to the ease of optimizing the platform for each species as well as its capacity to produce thousands of short, high quality polymorphic loci using a custom analytical pipeline [35–37]. Four methods of complexity reduction were tested in *R. clamitans* (data not presented) and double digestions with *Pst*I-*Sph*I method was selected.

DNA samples were processed in digestion/ligation reactions principally as per Kilian et al. [27] but replacing a single *Pst*I-compatible adaptor with two different adaptors corresponding to two different Restriction Enzyme (RE) overhangs. The *Pst*I-compatible adapter was designed to include Illumina flowcell attachment sequence, sequencing primer sequence and “staggered”, varying length barcode region, similar to the sequence reported by Elshire et al. [29]. The reverse adapter contained flowcell attachment region and *Sph*I-compatible overhang sequence. Only “mixed fragments” (*Pst*I-*Sph*I) were effectively amplified in 30 rounds of PCR using the following reaction conditions: 94̊ C for 1 min then 30 cycles of 94 °C for 20 s, 58 °C for 30 s, 72 °C for 45 s, and 72 °C for 7 min. After PCR, equimolar amounts of amplification products from each sample of the 96-well microtiter plate were bulked and applied to c-Bot (Illumina) bridge PCR followed by sequencing on Illumina Hiseq2500. The sequencing (single read) was run for 77 cycles.

Sequences generated from each lane were processed using proprietary DArT analytical pipelines (PL). In the primary pipeline the Fastq files were first processed to filter away poor quality sequences, such as those with reproducibility below 90 %, read depth lower than 3.5 for SNPs or 5 for presence-absence markers, and applying more stringent selection criteria to the barcode region compared to the rest of the sequence. In that way the assignments of the sequences to specific samples carried in the “barcode split” step were very reliable. No samples were dropped due to low coverage across loci but individual sequences were removed if they did not meet the above criteria. Approximately 2,500,000 sequences per barcode/sample were identified and used in marker calling. The average read depth across loci was 9.2 reads per individual per locus for reference alleles and 6.5 for SNP alleles. Finally, identical sequences were collapsed into “fastqcoll files”. The fastqcoll files were “groomed” using DArT PL’s proprietary algorithm which corrects low quality base from singleton tag into a correct base using collapsed tags with multiple members as a template. The “groomed” fastqcoll files were used in the secondary pipeline for DArT PL’s proprietary single nucleotide polymorphism (SNP) and SilicoDArT (presence/absence of restriction fragments in representation; PA markers) calling algorithms (DArTsoft14). For SNP calling all tags from all libraries included in the DArTsoft14 analysis are clustered using DArT PL’s C++ algorithm at the threshold distance of 3, followed by parsing of the clusters into separate SNP loci using a range of technical parameters, especially the balance of read counts for the allelic pairs. Additional selection criteria were added to the algorithm based on analysis of approximately 1000 controlled cross populations. Testing for deviations from Hardy-Weinberg equilibrium of alleles in these populations facilitated selection of technical parameters to effectively discriminate true allelic variants from paralogous sequences. In addition, multiple samples were processed from DNA to allelic calls as technical replicates and scoring consistency was used as the main selection criteria for high quality/low error rate markers. Calling quality was assured by high average read depth per locus (average across all markers was over 30 reads/locus).

### Marker selection

Our goal was to isolate single nucleotide polymorphisms (SNP) and presence-absence (PA) markers that are sex-linked in *R. clamitans*. For SNP loci, we define the “reference allele” as the allele that was sequenced most often in our data. For sex-linked markers in an XX-XY sex determining system, reference alleles will be those found on the X-chromosome. Here, “SNP alleles” are those which show polymorphisms relative to the reference allele. In an XX-XY system, SNP alleles should be alleles associated with the Y-chromosome and, as a corollary, should be on or near the male-determining region if the allele is tightly Y-specific. Higher degrees of recombination may be expected between sex chromosomes in endothermic species with homomorphic sex chromosomes [14, 25, 38]. In an XX-XY system if the two sex chromosomes recombine, SNP alleles should occasionally appear on the X chromosome, particularly in more distal regions of the sex chromosomes [25, 39]. If this occurs, then some males might be homozygous for SNP alleles at particular loci. Because of this, females could also be heterozygous, exhibiting a copy of the SNP allele. The probability of a female being homozygous for a SNP allele is low.

A previous study on inheritance patterns of an allozyme suggested an XX-XY system in *R. clamitans* [40]. While we assumed an XX-XY system, we also searched for loci showing a ZZ-ZW pattern. For evaluating loci associated with an XX-XY system, we targeted SNP loci that maximized female homozygosity at the reference allele, maximized male heterozygosity, and which minimized homozygosity for the SNP allele in both sexes, but particularly for females. Specifically, we kept loci where frequencies of female homozygosity for the reference allele were at least 80 %, homozygosity at the SNP allele were at most 10 %, and heterozygosity were no more than 20 %. For males, we kept loci where frequencies of homozygosity for the reference allele were at most 10 % and heterozygosity were at least 75 %. This allowed sex-linked markers to show higher degrees of SNP allele homozygosity for males if recombination were occurring. We also only included SNP loci which were sequenced for at least 90 % of each sex. For PA markers we selected loci which had restriction fragments sequenced in at least 90 % of one sex and not sequenced in at least 90 % of the other sex. For both SNP and PA markers, we analyzed tadpole and adults data separately and compared results between these groups afterwards. We took a similar but opposite approach for targeting loci with a ZZ-ZW system.

To assess the genetic association between each locus and phenotypic sex, we performed Cochran-Armitage tests on the adult samples with the “independence_test” function in the R package ‘coin’. We also used Hamming Distance matrices with a custom R script to estimate how well the combined markers perform in predicting phenotypic sex. Hamming Distance calculates the number of pairwise differences between all individuals across all loci. For all sex-linked loci which met our criteria and which had statistically significant associations with phenotypic sex, we used NCBI BLAST [41] to discover homologies of sex-linked SNPs markers. Specifically, we searched the “nr” database with the default settings in NCBI’s blastn suite. We searched using both the REF and SNP alleles and only report blastn hits related to sexual development or sex determination and those with e-values of 1.5 or below.

### Estimating random sex-linkage

Because high-throughput sequencing provides thousands of markers, we wanted to estimate that probability that some of these markers might show random associations with sex. This could be particularly problematic if small sample sizes are used to develop markers [23]. The following formula describes the probability of a locus being sex-linked by chance.

$$ {P}_i={0.5}^n $$

Here *P* is the probability that a given locus, *i*, is sex-linked. The probability that either a female is homozygous at a given locus or a male or heterozygous is 0.5. The number of individuals sequenced at the locus is *n*. After sequencing, we multiplied *P* by the number of high-quality SNPs produced to estimate how many SNPs were expected to exhibit a sex-linked pattern by chance. Using this calculation, we estimated the estimated number of expected sex-linked markers produced by chance across a range of sample sizes and loci for comparisons with other studies. We also estimated the number of expected random sex-linked loci for both the tadpole samples as well as the adult samples used here.

## Results

Prior to filtering, we sequenced 120,829 SNP loci and additional 92,604 PA markers. After filtering, we retained 42,772 SNP loci representing 2,135,560 bp, approximately 0.2 % of *R. clamitans*’ genome (~11.9 pg diploid DNA content) [42]. Our filtering also yielded 27,850 PA markers, equaling 1,174,376 bp which is approximately 0.1 % of the genome. Based on our selection criteria, a total of 13 SNPs were identified to assort to males (Table 2) and these SNPs were accessioned to NCBI’s dbSNP database (NCBI accession PRJNA326426, ss# 2019323440-2019323452). For the 13 sex-linked SNP loci, the average read depth across the loci is 12.7 (range 7.2–20.6) on the reference allele and 9.0 (range 5.0–13.1) on the SNP allele. SNP locus RaclCT001 was found to be perfectly sex-linked in all stages, and if we had the linkage data, this locus might possibly be linked or almost completely linked with the sex determining locus. RaclCT002 was perfectly sex-linked in tadpoles and performed almost as well in adults with 4 % (*n* = 2) of males exhibiting the genotype most commonly found in females at this one locus. Nine SNP loci were independently identified in the tadpole and adult samples. Two loci were identified separately each in the tadpole samples and the adult samples (Table 2). Cochran-Armitage tests verified significant SNP allelic association with phenotypic sex for all loci (*χ* ^2^ = 33.07–74.84, all *p* < 9.00 e -09). Each marker was homozygous for the reference allele in females and heterozygous in males. This confirms an XX-XY sex determining system in *R. clamitans* as previously suggested [37].

**Table 2:** Sex-linked loci, including reference (REF) and SNP allele sequences with SNP positions bolded

			Tadpoles						Adults					
			Reference allele homozygous proportion		SNP allele homozygous proportion		Heterozygous proportion		Reference allele homozygous proportion		SNP allele homozygous proportion		Heterozygous proportion	
Locus	Allele	Sequence	Female	Male	Female	Male	Female	Male	Female	Male	Female	Male	Female	Male
RaclCT001	REF	TGCAGCCAACATGTGTTTATGT**G**CTTTGTTCAGCATG	1.00	0.00	0.00	0.08	0.00	0.92	1.00	0.00	0.00	0.02	0.00	0.98
	SNP	TGCAGCCAACATGTGTTTATGT**A**CTTTGTTCAGCATG												
RaclCT002	REF	TGCAGCGAGAACATTT**C**GGCATG	1.00	0.00	0.00	0.00	0.00	1.00	1.00	0.04	0.00	0.00	0.00	0.96
	SNP	TGCAGCGAGAACATTT**T**GGCATG												
RaclCT003	REF	TGCAGACAGTTGATGACTT**C**TGCGCACTGTGTCCTTCAGCATG	1.00	0.00	0.00	0.23	0.00	0.77	1.00	0.06	0.00	0.11	0.00	0.83
	SNP	TGCAGACAGTTGATGACTT**T**TGCGCACTGTGTCCTTCAGCATG												
RaclCT004	REF	TGCAGTAGGCATTGGTGATTCATT**G**ATTGTTTTATGCATG	0.92	0.00	0.00	0.00	0.08	1.00	0.96	0.13	0.00	0.02	0.04	0.85
	SNP	TGCAGTAGGCATTGGTGATTCATT**A**ATTGTTTTATGCATG												
RaclCT005*^a^	REF	TGCAGACAAACTCATGCTGTGTCTAATCACAGCACAGGTCAGAGT**G**GGGCATGTGCATG	0.75	0.00	0.00	0.08	0.25	0.92	0.91	0.07	0.00	0.04	0.09	0.89
	SNP	TGCAGACAAACTCATGCTGTGTCTAATCACAGCACAGGTCAGAGT**A**GGGCATGTGCATG												
RaclCT006^a^	REF	TGCAGTGTTATTGCAT**C**ATAGGAGCATG	0.92	0.31	0.00	0.00	0.08	0.69	0.91	0.07	0.00	0.04	0.09	0.89
	SNP	TGCAGTGTTATTGCAT**T**ATAGGAGCATG												
RaclCT007^a^	REF	TGCAGCTCAGT**C**TCTCCGGCCTCTGTGTGTCCTGTCCTTGACAGCATG	1.00	0.38	0.00	0.00	0.00	0.62	0.96	0.07	0.00	0.13	0.04	0.80
	SNP	TGCAGCTCAGT**A**TCTCCGGCCTCTGTGTGTCCTGTCCTTGACAGCATG												
RaclCT008	REF	TGCAGATGAGGATGTACTGGCTTCACTGGCTT**C**AATTACTGCATG	0.92	0.00	0.00	0.00	0.08	1.00	0.87	0.09	0.00	0.04	0.13	0.87
	SNP	TGCAGATGAGGATGTACTGGCTTCACTGGCTT**T**AATTACTGCATG												
RaclCT009^a^	REF	TGCAGCTGGGTCTGAT**C**CCACAAGATCCTTCATCGTCGCATG	1.00	0.38	0.00	0.00	0.00	0.62	0.91	0.06	0.04	0.13	0.04	0.81
	SNP	TGCAGCTGGGTCTGAT**T**CCACAAGATCCTTCATCGTCGCATG												
RaclCT010	REF	TGCAGTTTTTTCTCACAAT**G**CAGCAGCATG	0.92	0.00	0.00	0.08	0.08	0.92	0.90	0.07	0.00	0.13	0.10	0.80
	SNP	TGCAGTTTTTTCTCACAAT**A**CAGCAGCATG												
RaclCT011*^a^	REF	TGCAGCTCACTCT**G**TACAGATTCCCTGCATGAGATCGGAAGAGCGGTTCAGCAGGAATGCCGAGACCGA	0.75	0.31	0.00	0.00	0.25	0.69	0.87	0.09	0.00	0.09	0.13	0.81
	SNP	TGCAGCTCACTCT**C**TACAGATTCCCTGCATGAGATCGGAAGAGCGGTTCAGCAGGAATGCCGAGACCGA												
RaclCT012^	REF	TGCAGCCATGTG**G**CTAATTAGAAGGCTGGAGGCTGCAAGTTTCTAGGCATG	0.92	0.00	0.00	0.08	0.08	0.92	0.74	0.04	0.00	0.11	0.24	0.85
	SNP	TGCAGCCATGTG**A**CTAATTAGAAGGCTGGAGGCTGCAAGTTTCTAGGCATG												
RaclCT013^	REF	TGCAGCT**G**TTTTTGCCACAAAGTGCATG	0.92	0.00	0.00	0.15	0.08	0.85	0.61	0.02	0.13	0.15	0.26	0.83
	SNP	TGCAGCT**T**TTTTTGCCACAAAGTGCATG												

*Markers were identified originally in the adult samples, but not the tadpole samples. Consequently tadpole samples show lower levels of female homozygosity for the reference allele and males show lower levels of heterozygosity

^a^Male tadpoles from pond Forest5 are predominantly or entirely homozygous at the reference allele, possibly indicating geographic variation in sex-linkage at this locus. Without these male tadpoles, these markers show higher sex linkage

^Markers were identified originally in the tadpoles samples, but not the adult samples. Consequently adult samples show lower levels of female homozygosity for the reference allele

**Fig. 3:**
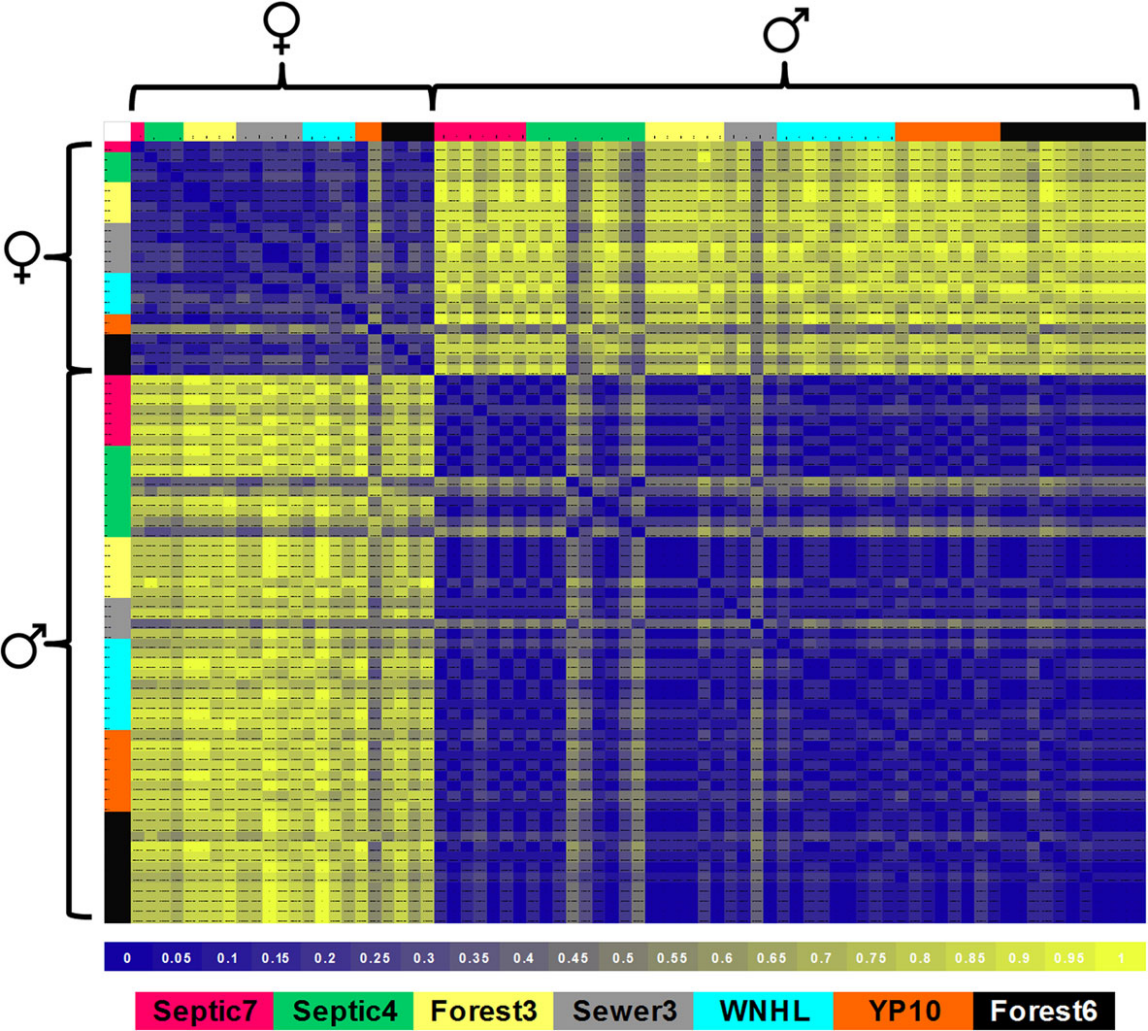
Hamming Distance matrix illustrating proportional differences in 13 sex-linked SNP markers across all analyzed adults. Sex-linked SNPs were originally identified in both lab-reared tadpoles and wild-caught adults. Hamming Distance calculates the number of pairwise differences among all individuals at these loci. Values closer to zero (*blue*) signify high similarity whereas values closer to one (*yellow*) are more dissimilar across the thirteen loci. Colored regions at the *top* and at the *left* correspond to source ponds. The matrix is clustered by phenotypic males and females, as indicated by symbols at the *left* and *top*. One phenotypic female is on average 52 % dissimilar from all other females, showing an ambiguous genotypic sex across the 13 loci. Similarly, four males are on average 36–49 % dissimilar from other males

**Fig. 4:**
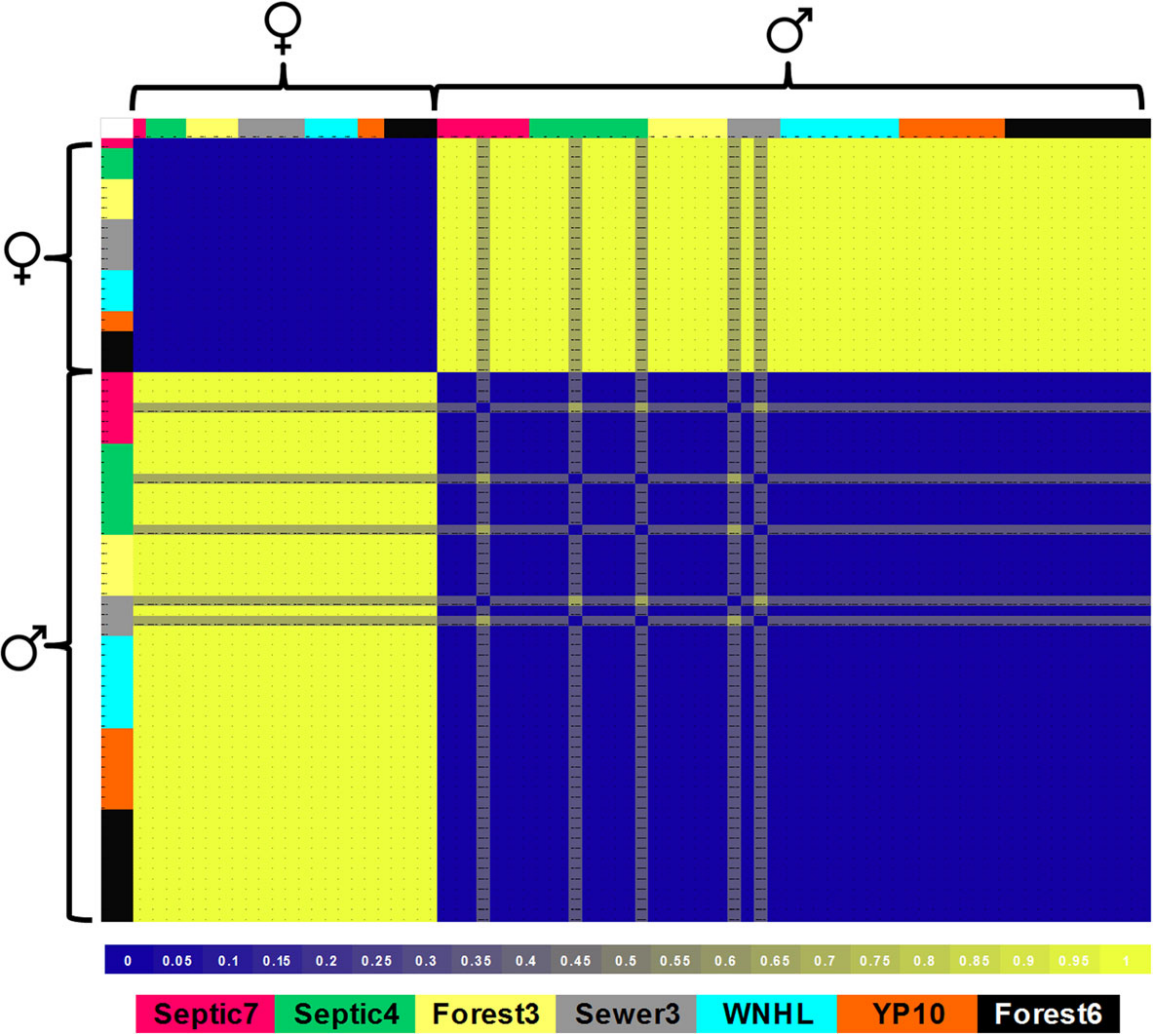
Hamming Distance matrix illustrating proportional differences across all analyzed adults using only the three most sex-linked SNP loci. Values closer to zero (*blue*) signify high similarity whereas values closer to one (*yellow*) are more dissimilar across the 13 loci. Colored regions at the *top* and at the *left* correspond to source ponds. At these three loci, all females show perfect concordance between genotypic and phenotypic sex. Most males also show perfect concordance between genotypic and phenotypic sex. Five males are discordant at only one of three loci

Hamming’s Distances calculating pairwise differences between all 13 SNP loci (Fig. 3) for all adults showed that females are on average 21 % dissimilar (±1.6 SE, min 14 %, max 52 %) from each other and males are on average 16 % dissimilar (±2.2 SE, min 9 %, max 49 %). A large part of this variation is due to a single female and five males (T0123777 from Sewer3 and T012338, T012340, T012373, and T012374 from Septic4). These individuals are discordant across multiple loci and, notably, many of the same loci across individuals. However, when looking at the three loci (RaclCT001, RaclCT002, RaclCT003) showing the highest degree of sex-linkage (Fig. 4), all adult females show perfect congruence between phenotypic and genotypic sex at all three loci. For males, Hamming’s Distances at the top three loci (Fig. 4) show that males are on average 6 % dissimilar (±1.0 SE, min 0.03 %, max 35 %). Using only these three loci, 91 % (*n* = 49) adult males show perfect congruence between phenotypic and genotypic sex at all three loci. The other 9 % (*n* = 5) of adult males are only discordant at only one locus (*n* = 2 at RaclCT002, *n* = 3 at RaclCT003). These three loci provide further evidence that the individuals previously mentioned which display multiple loci more commonly seen in the opposite sex are not sex-reversed but may have inherited a series of linked loci during a recombination event in a parental germ line.

Eight PA markers (Table 3) also showed significant sex-linkage (Cochran-Armitage; (*χ* ^2^ = 44.42–55.46, all *p* < 1.05 e -11). For the PA markers, four loci met our criteria only in tadpoles and four only in adults, with no locus meeting our criteria in both life stages. All PA marker restriction fragments were characterized by their presence in male samples and absence in female samples. Hamming’s Distances (Additional file 2: Figure S1) showed that these eight PA loci in adults showed that females are on average 11 % dissimilar (±2.1 SE, min 51 %, max 5 %) from each other and males are on average 11 % dissimilar (±1.5 SE, min 0.04 %, max 64 %).

**Table 3:** Sex-linked markers and sequences for eight presence-absence (PA) loci in *R. clamitans*

		Proportion with restrction fragment sequenced			
Locus	Sequence	Tadpoles		Adults	
		Females	Males	Females	Males
RaclCT014^ab^	TGCAGTGTCTCTGAGGGTTTACTGGTGATCCAGCGCATG	0.27	0.58	0.00	0.92
RaclCT015^ab^	TGCAGCATATGTGCGTACGGTCGGCGGGAAGGGGTTAAGCTATGTCCAGTGCCCTGCATG	0.00	0.58	0.09	0.94
RaclCT016^ab^	TGCAGCTCAGTATCTCCGGCCTCTGTCTGTCCTGTCCTTGACAGCATG	0.00	0.62	0.04	0.92
RaclCT017^ab^	TGCAGAAGTGCAGTGCATTGCTGTATGATTGGCCAAAGCATG	0.30	0.60	0.09	0.96
RaclCT018^c^	TGCAGCAAGAGGTGAAAACAACCGCTGTTGGCAGCATG	0.00	1.00	0.04	0.89
RaclCT019^c^	TGCAGTGCTTGAGATGGATCACACAGTGTGATCCATCTCAAAAACTGCGACTGTTGCATG	0.00	1.00	0.05	0.89
RaclCT020^c^	TGCAGGCTGCAAAGAAGAAACGAGAAAGCTGCATG	0.00	1.00	0.13	0.94
RaclCT021^c^	TGCAGCATTGCAGTGCATTGTTGTCTGATGATTGGGCAAGCATG	0.00	1.00	0.04	0.94

^a^These loci were originally identified as sex-linked with adult specimens but not tadpoles

^b^These markers are almost entirely unsequenced in male tadpoles from Forest 5. Restriction fragments for these loci were sequenced in all other male tadpoles

^c^These loci were originally identified as sex-linked with tadpole specimens but not adults. While perfectly sex-linked in tadpoles, they are less sex-linked in adults

*BLAST analysis*: Three SNP markers but no PA markers had BLAST sequence homologies (Additional file 3: Table S2) to sex-related sequences. Two SNP loci had sequence homology, as identified by our BLAST search, to sex chromosomes in other taxa including matches to chimpanzee (*Pan troglodytes*) and human X-chromosome (RaclCT006) as well as chicken (*Gallus gallus*) Z-chromosomes (RaclCT013). While these homologies are on hominid and bird sex chromosomes, possibly indicating conserved genomic regions important in sexual development throughout tetrapods, these sequence homologies, to our knowledge, do not have known functions. Of particular interest is locus RaclCT002, which showed the highest sex-linkage in tadpoles and second highest sex-linkage in adults. RaclCT002 exhibited sequence similarity (Additional file 3: Table S2) to Dmrt1 in three other Ranid frogs (*Rana nigromaculata*, *Rana rugosa*, and *Rana chensinensis*) as well as a minnow (*Cyrpinodon variegatus*). As a preliminary analysis, we translated RaclCT002 into an amino acid sequence using ExPASy and searched for amino acid homology with UniProt BLAST. This preliminary analysis showed 100 % homology over seven amino acids (MPKCSRC) with the DM binding domain in multiple taxa including the Japanese wrinkled frog (*Rana rugosa*) and the Chinese brown frog (*Rana chensinensis*) as well as several fishes including rainbow trout (*Oncorhynchus mykiss*), medaka (*Oryzias latipes*), and zebra fish (*Danio rerio*).

**Fig. 5:**
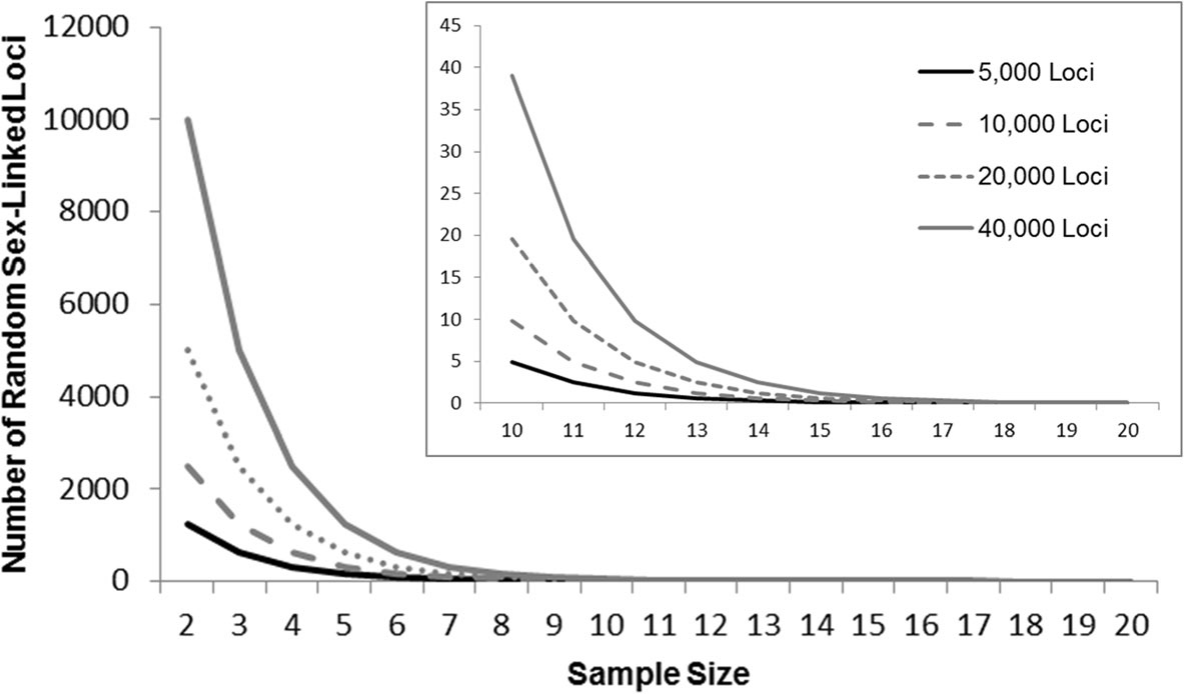
The modeled number of loci that are expected to be sex-linked by chance with varying sample sizes and number of polymorphic loci assessed. *Inset* is the same plot but focused on the latter half of sample sizes

*Estimating Random Sex-Linkage*: Across a range of sample sizes and loci, 13–16 individuals are necessary to minimize the probability of producing less than one spurious sex-linked marker (Fig. 5, Additional file 2: Figure S2). For our 25 tadpoles, *P*_*i*_ the probability that a single locus exhibited a sex-linked pattern by chance was 2.98 × 10^−8^ and, of the 42,772 loci, 0.001 are expected to spuriously showed sex-linkage, and therefore random sex-linked markers in the tadpoles is highly unlikely. However, because our tadpole samples were represented by only six clutches we also performed a more conservative analysis were *P*_*i*_ for a sample size of 12 (one male and one female per clutch) is 2.4 × 10^−4^. With just a single male and female from each clutch, it is possible that approximately 10.4 loci might show spurious sex-linkage by chance. The analyses for tadpoles using either a sample size of 25 or 12 indicate that some loci may be sex-linked by chance. For the 72 adults, *P*_*i*_ is 2.12 × 10^−22^ and the probability that one locus spuriously showed sex-linkage was 9.06 × 10^−18^. Given our sample size, this probability indicates that it is highly unlikely to identify any sex-linked markers by chance in our adult samples.

## Discussion

Thirteen of 42,772 SNPs showed sex-linkage in *R. clamitans*, two of which are perfectly sex-linked or almost perfectly aligned to phenotypic sex assignment. In addition, our analysis produced eight sex-linked markers associated with the presence or absence of restriction fragments (PA markers). These loci represent the first sex-linked DNA markers in any New World amphibian. Furthermore, all of our markers conform to an XX-XY system in *R. clamitans* as previously inferred through inheritance differences in a sex-linked allozyme locus [40]. Our results reinforce the usefulness of methods incorporating genome complexity reduction and high throughput sequencing, like DArTseq or RADseq, for studying sex determination and identification of sex determining modes in non-model organisms, without a-priori sequence information [23].

Of particular interest is the homology RaclCT002 shows to Dmrt1. In Europe, Dmrt1is either sex-linked or likely important in sexual differentiation in several Hylidae, *Rana temporaria*, and *Bufo viridis* [43, 44]. Furthermore, Dmrt1is an important gene in sexual differentiation in the Japanese wrinkled frog (*Rana rugosa*), despite being autosomal [45]. Given the Dmrt family’s ubiquitous role in sex determination and differentiation throughout Metazoa [46, 47] the homology of RaclCT002 to several other Ranid Dmrt1sequences provides further support for Dmrt1is important in amphibian sex determination. However, due to the relatively short sequence length of this marker, more work is needed to fully characterize this gene and its functionality in *R. clamitans*.

Of the 77 adults within the study, a single female and five males are consistently discordant at RaclCT003-008, RaclCT010, and RaclCT011. One explanation is that these loci are linked and lie on region of the Y-chromosome experiencing recombination with the X-chromosome, which can even frequently occur in species like humans [48]. While these loci typically diagnose sex accurately in the rest of our samples, it is important to note possible linkage might produce ambiguous genetic sexes for certain individuals. Despite this concern, these six individuals showed concordance between phenotypic and genotypic sex in at least two of the three top ranked loci. This pattern suggests that while all the identified sex-linked loci can be diagnostically useful, RaclCT001-003 may be the most reliable in cases of ambiguous sexing.

The markers developed here are predominantly robust to geographic variation at the scale of up to 52 km as well as substantial land use variation among source ponds (Fig. 1). Interestingly though, tadpoles from Forest5, the northern-most pond, show no sex linkage at four SNP and four PA loci (Tables 2 and 3). No other populations show this. While we did not sample adults from Forest5 and the tadpole samples only represent two wild-collected clutches, this pattern could result from geographic variation in sex-linkage at some loci. In the European common frog, (*Rana temporaria*), sex-linked microsatellite markers originally developed for populations in Finland [14, 30] show decreasing levels of sex linkage with decreasing latitude in neighboring Sweden [49, 50]. While our study sites represent a much narrower geographic distribution than the latitudinal gradient in Sweden, it is possible that sex chromosome recombination or turnover [38] diminishes sex-linkage at certain loci. Given the broad range of *R. clamitans* across much of eastern North American (Fig. 1a), it will be necessary to explore potential geographic variation in sex-linkage across these loci.

Recently, Brelsford et al. [27] failed to identify sex-linked markers using RADseq in *R. temporaria*, concluding the absence of genetic sex determination in the population where the parents were sampled from. However, this study was attempting to produce a linkage map and therefore only used offspring and parents from a single mating. Brelsford et al. [26] suggested sampling from a broader geographic distribution and with more genetic variation may be useful for identifying sex-linked loci. Furthermore, Brelsford et al. [44] used a GBS approach on four species of European Hylid frogs. With four males and four females of each species, they found between 17 and 54 putative sex-linked SNP loci for each species out of between 4043 and 10,211 possible polymorphic sites [41]. However, our analysis (Fig. 5, Additional file 2: Figure S2), indicates that the sample sizes used for these Hylids is likely to yield 20–40 sex-linked markers by chance. This means that many of these Hylid sex-linked markers are likely false positives, without validation. Our results suggest that GBS-type methods should target multiple individuals from a given location and should focus on multiple, disparate populations. This is particularly true in taxa like many amphibians which may be expected to undergo sex reversal [14], sex chromosome recombination [38, 51], and sex chromosome turnover [7]. Studies wishing to employ similar methods should sample 13 or more individuals with a relatively even mixture of both sexes to minimize the probabilities of false-positives, though the number of samples needed may also depend on the amount of polymorphism present in a population which will likely vary across species.

Importantly, nine SNP loci were independently discovered in both the lab-reared tadpole samples as well as the wild-caught adults, increasing our confidence in these markers. Of the remaining four SNP loci, one (RaclCT005) was discovered only in adults and was possibly not discovered in tadpoles due to geographic variation at Forest5 where the locus was not sex-linked. The other marker (RaclCT011), identified only from the adult samples, was itself weakly sex-linked among the adult samples with relatively high level of female heterozygosity and lower male heterozygosity than other markers and might therefore be less reliable (Table 2). The two loci (RaclCT012, -013) found only in the tadpole samples show high levels of female heterozygosity in adults (Table 2), making them less reliable in sex identification. Our analysis indicates that these two loci may be sex-linked here by chance due to the smaller tadpole sample size and provide further support for using larger sample sizes in future studies.

Sex reversal would be diagnosed by discordance between the phenotypic sex and the majority of sex-linked markers, particularly RaclCT001-003. We did not detect any clear evidence of sex reversal in our samples. This may be due to a relatively low frequency of sex reversal compared to the sample size from each source pond. In a study of sex reversal in the European common frog (*R. temporaria*), Alho et al. [14] used 79 adults collected over a three year period at a single site. They found that 5 % of genetic females (*n* = 55 total) had a male phenotype. Here we used 77 adults collected from seven populations, and all but five individuals were collected during the same year. However, the maximum number of females from any pond was five (μ = 3.6, SE = 0.5) and the maximum number of males was 11 (μ = 7.4, SE = 0.8). If sex reversal is occurring in *R. clamitans*, it is possible that our sample sizes at any given site were too low to detect it. A richer evaluation of sex reversal in this system must use larger sample sizes at a given population, both in adults as well as larval and metamorphosing individuals.

Over the past two decades, *R. clamitans* has emerged as a valuable model species for the study of endocrine disruption in both field studies [16, 17, 19, 52, 53] as well as experimental research [54, 55]. In laboratory experiments with other amphibian species, only two studies have been able to show true sex reversal in response to chemicals using sex-linked markers [3, 13]. No study has yet to assess whether endocrine disruption in wild amphibians also results in sex reversal. The markers developed here will allow a more thorough investigation as to whether *R. clamitans* populations are experiencing sex reversal and at what frequencies.

## Conclusions

Methods, like DArTseq or RADseq, combining genome complexity reduction with high throughput sequencing, are valuable options for studying the genetic basis of sex determination, gaining direct sequence insight, and providing opportunities to discover novel genes and sequences in sex determining and sex differentiating pathways. With DArTseq we discovered the first sex-linked markers in a North American amphibian and elucidated a putative sex determining gene (Dmrt1). The number of markers developed here is impressive given the small proportion of the genome (<0.5 %) analyzed and highlights the usefulness of this approach to identifying sex-linked markers. While these markers will need to be tested throughout the *R. clamitans* range, they provide a novel tool for studying sex determination and sex reversal in wild populations of this species. Our approach also provides information on minimizing probabilities of detecting false-positives when planning similar future studies. DArTseq and comparable methods promise to advance our understanding of the evolution of sex determination in amphibians and vertebrates more broadly [5]. The markers we developed provide an important step in understanding patterns of sex reversal and sexual differentiation variation throughout the *R. clamitans* range and provide early insight into the evolution of sex determination in amphibians.

## Additional files

Additional file 1: Table S1. Specimen data including Yale Peabody Museum tag numbers for adult specimens used in this study. (XLSX 14 kb)
Additional file 2: Figures S1 and S2. Hamming distance matrix for PA markers and simulation results for the number of sex-linked markers expected by chance with varying numbers of loci evaluated and samples used. (DOCX 448 kb)
Additional file 3: Table S2. BLAST results for sex-linked markers including locus name, allele, homology, and e-value. (XLSX 11 kb)

## Abbreviations

*DArTseq*: Diversity Arrays Technology sequencing
*GBS*: Genotyping-by-sequencing
*GSD*: Genotypic sex determination
*RADseq*: Restriction site-associated DNA sequencing
SNP: Single nucleotide polymorphism
