# Supplementary material for: Sex-linked markers in the North American green frog (Rana clamitans) developed using DArTseq provide early insight into sex chromosome evolution
Source: BMC Genomics. 2016 Oct 28;17:844. doi: 10.1186/s12864-016-3209-x (PMC5084323; doi:10.1186/s12864-016-3209-x)
Supplement: Additional file 2: Figures S1 and S2. — Hamming distance matrix for PA markers and simulation results for the number of sex-linked markers expected by chance with varying numbers of loci evaluated and samples used. (DOCX 448 kb) [file 12864_2016_3209_MOESM2_ESM.docx]

Sex-linked markers in the North American green frog (*Rana clamitans*) developed using DArTseq elucidate Dmrt1 as a putative sex-determining gene and provide early insight into sex chromosome recombination

Max R. Lambert, David K. Skelly, Tariq Ezaz

Additional file


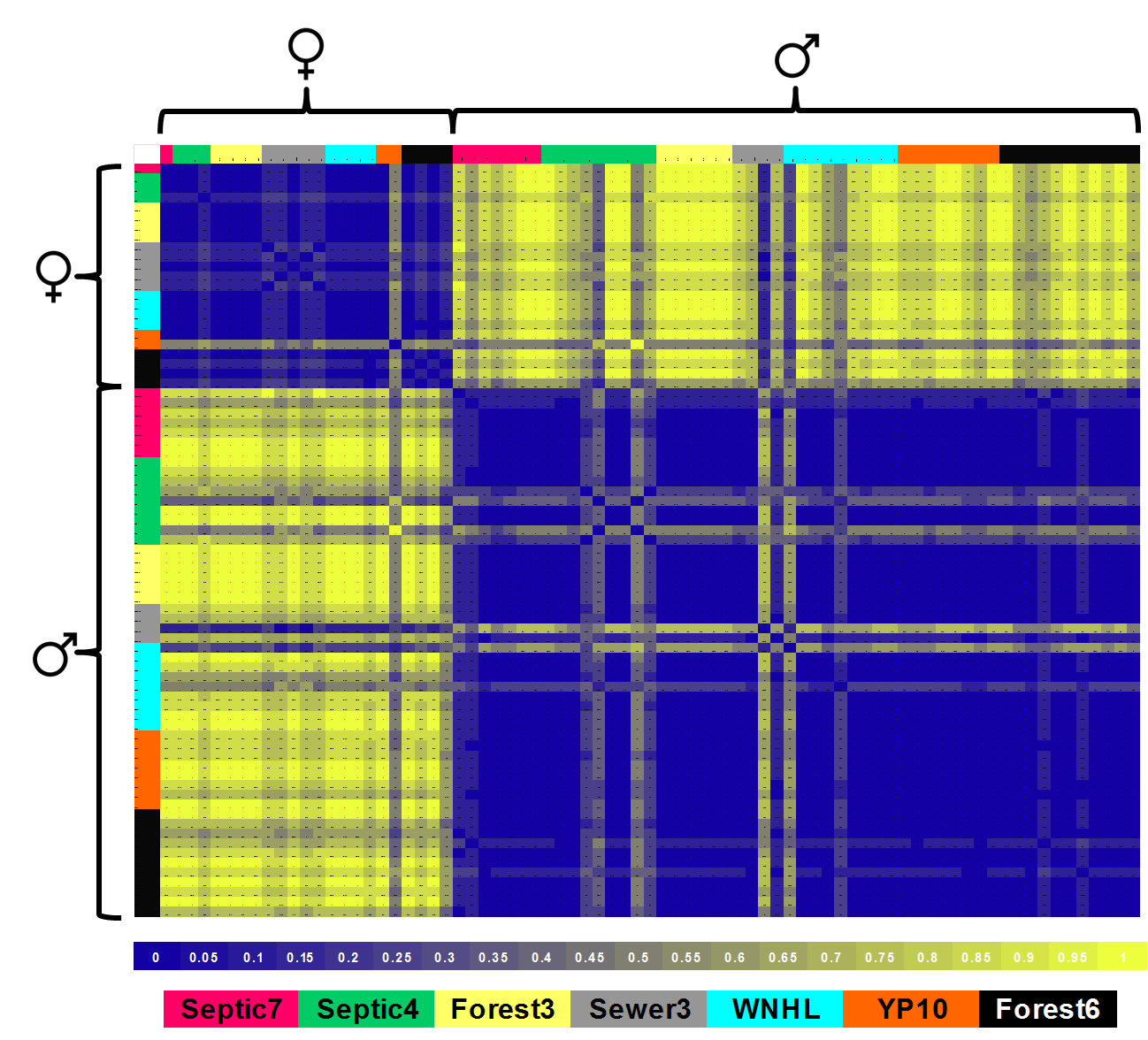


Figure S1: Hamming Distance matrix illustrating proportional differences in eight sex-linked Presence-Absence (PA) markers across all analyzed adults. Sex-linked PA markers were originally identified in both lab-reared tadpoles and wild-caught adults. Hamming Distance calculates the number of pairwise differences among all individuals at these loci. Values closer to zero (blue) signify high similarity whereas values closer to one (yellow) are more dissimilar across the thirteen loci. Colored regions at the top and at the left correspond to source ponds. The matrix is clustered by phenotypic males and females, as indicated by symbols at the left and top.


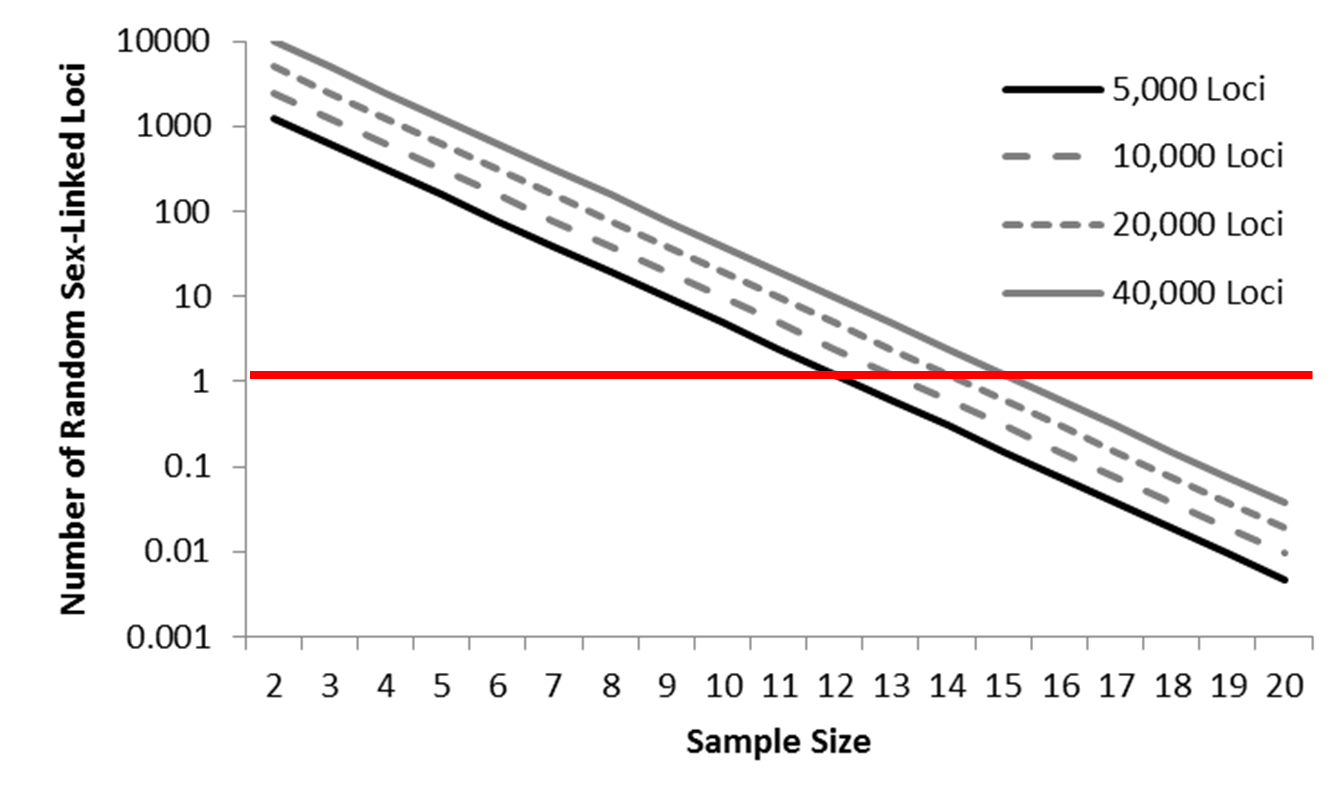


Figure S2: The modeled number of loci that are expected to be sex-linked by chance with varying sample sizes and number of polymorphic loci assessed. This is the same plot at Figure 5 but on a log scale. The horizontal red line indicates the sample size at which a single locus is expected to be sex-linked by chance for different numbers of loci assessed.
